# Improvements in Gut Microbiome Composition Predict the Clinical Efficacy of a Novel Synbiotics Formula in Children with Mild to Moderate Atopic Dermatitis

**DOI:** 10.3390/microorganisms11092175

**Published:** 2023-08-28

**Authors:** Chi Tung Choy, Pui Ling Kella Siu, Junwei Zhou, Chi Ho Wong, Yuk Wai Lee, Ho Wang Chan, Joseph Chi Ching Tsui, Claudia Jun Yi Lo, Steven King Fan Loo, Stephen Kwok Wing Tsui

**Affiliations:** 1 Microbiome Research Centre, BioMed Laboratory Company Limited, Hong Kong; 2 Hong Kong Institute of Integrative Medicine, Faculty of Medicine, The Chinese University of Hong Kong, Hong Kong; 3 Dermatology Centre, CUHK Medical Centre, The Chinese University of Hong Kong, Hong Kong; 4 School of Biomedical Sciences, Faculty of Medicine, The Chinese University of Hong Kong, Hong Kong; 5 Centre for Microbial Genomics and Proteomics, The Chinese University of Hong Kong, Hong Kong; 6 Hong Kong Bioinformatics Centre, The Chinese University of Hong Kong, Hong Kong

**Keywords:** atopic dermatitis, gut microbiome, probiotics

## Abstract

Atopic dermatitis (AD) is a common chronic inflammatory skin disease with a significant association with various type-2 inflammation-related comorbidities. Ongoing research suggests the crucial involvement of gut microbiome, especially in childhood onset AD, and hence, probiotics have emerged as a potential non-steroid-based therapeutics option to complement existing AD management plans. In order to delineate the impact of probiotics in the gut microbiome of pediatric AD patients from southern China, targeted 16S rRNA sequencing and thorough bioinformatic analysis were performed to analyze the gut microbiome profiles of 24 AD children after taking an orally administered novel synbiotics formula with triple prebiotics for 8 weeks. A notable improvement in Eczema Area and Severity Index (EASI) (*p* = 0.008) was observed after taking an 8-week course of probiotics, with no adverse effects observed. The relative abundances of key microbial drivers including *Bacteroides fragilis* and *Lactobacillus acidophilus* were significantly increased at week 8. We also found that the positive responsiveness towards an 8-week course of probiotics was associated with improvements in the gut microbiome profile with a higher relative abundance of probiotic species. Over-represented functional abundance pathways related to vitamin B synthesis and peptidoglycan recycling may imply the underlying mechanism. In summary, our study suggests how the gut microbial landscape shifts upon probiotic supplementation in AD children, and provides preliminary evidence to support targeted probiotic supplementation for the management of childhood AD.

## 1. Introduction

Atopic dermatitis (AD) is a common chronic inflammatory skin disease with both atopic and nonatopic comorbidities [1] including asthma, rhinitis; and psychiatric, autoimmune, and cardiovascular diseases [2,3,4,5,6,7,8,9,10,11,12,13,14]. The incidence of childhood eczema has been continuously rising, especially in developed countries [14,15,16]; the lifetime eczema prevalence among children could be as high as 30% in China [17,18,19,20,21]. The “hygiene hypothesis” postulates that the lifestyles of people in industrialized countries, together with a decline in the infectious burden, have been associated with an increase in allergic and autoimmune diseases [22,23]. In recent years, this hypothesis has gained increasing attention from the scientific community to explain the time trend. Although the exact cause of AD is not fully understood, it is known to result from a complex interplay of genetic, environmental, and immunological factors [24]. With advances in sequencing technology, researchers have begun to investigate the relationship between the gut microbiome and pediatric AD, and it is clear that children with AD have a distinct gut microbiome composition compared to healthy children [25,26,27,28,29,30,31,32,33]. Specifically, children with AD have been found to harbor gut dysbiosis with altered levels of beneficial bacteria, such as *Bifidobacterium* and *Lactobacillus*, and enriched subspecies of *Faecalibacterium prausnitzii* [28,31,32,34]. These alterations could lead to dysfunction of the intestinal barrier [35] and dysregulation of the immune system [36], e.g., inhibiting Th1 and/or Th2 maturation [37,38,39]. Hence, current evidence suggests that appropriate microbial colonization in early life may lower the risk of developing allergic diseases [40] as described in the “hygiene hypothesis”, and the gut microbiome may contribute to the underlying mechanism to some extent.

Modulating the gut microbiome as a therapeutic strategy for AD has therefore been explored in the form of prebiotics, probiotics, fecal microbiota transplantation [41], and dietary interventions [42]. In particular, the World Allergy Organization has recommended probiotic use in infants at high risk of developing allergies [43]. However, study results are inconsistent and mixed, with some groups reporting the positive beneficial effect of probiotics in alleviating AD symptoms and mitigating the risk of AD, but others reporting no effect [44,45]. Despite the ongoing debate on the best practices of probiotic use in treating AD, a number of recent meta-analyses have pooled several clinical trials [46,47,48,49,50,51] and shown that certain probiotic strains [52,53,54], such as *Lactobacillus rhamnosus* and *Bifidobacterium lactis*, may be effective in reducing AD symptoms in children. In addition, mixed probiotic strains with a longer treatment duration have apparently demonstrated better effects in children aged > 1 year [46]. However, the specific mechanisms by which probiotics exert their effects are not yet fully understood, and further investigation is warranted to elucidate the optimal bacterial strains, administration dosages, and treatment durations for achieving the desired therapeutic outcomes.

Geography, lifestyle, and diet are some of the other well-known factors influencing gut microbiota composition [55,56]. Recent studies have also suggested that the efficacy of probiotics may differ based on the host’s gut microbiome composition [57]. In view of these, in our study, we investigated the efficacy of a novel synbiotics formula in southern Chinese children with AD, and the gut microbiome dynamics upon probiotics intervention. We believe that our pilot study could augment the current knowledge in respect of the role of gut microbiota in the management of AD in early childhood and provide insights into the underlying mechanism.

## 2. Materials and Methods

### 2.1. Subject Recruitment and Study Design

(1–10 years old) with mild to moderate AD were recruited prospectively from a community health survey through local skin patient association. All participants’ legal guardians provided informed consent. Exclusion criteria of the study were as follows: (1) history of adverse reaction to probiotics; (2) known overt bacterial infections in the skin; (3) premorbid medical conditions such as cardiovascular, liver, or renal dysfunction; or diabetes mellitus; (4) prior use of oral corticosteroids, oral antibiotics, other immunosuppressive drugs, or any preparation of oral herbal medicines for the treatment of AD in the past month; (5) prior diagnosis of AD, scabies, allergic contact dermatitis, or seborrheic dermatitis; and (7) prior use of anti-coagulant or anti-platelet drugs in the past month. All study participants underwent an initial screening by a board-certified dermatologist (S.K.F.L) to evaluate the severity of their atopic dermatitis. Stool specimens were obtained for subsequent sequencing analysis. Participants were permitted to continue their customary pharmacological or topical maintenance therapy for atopic dermatitis during the course of the study. Participants were followed up to confirm their compliance and collect fecal samples after 8 weeks. Age- and sex-matched historical control of deidentified AD patients from previous longitudinal studies without the use of probiotics were extracted from the database and used for comparison. The study was conducted in compliance with the ethical principles outlined in the Declaration of Helsinki and received approval from the Research Ethics Committee of the Hong Kong Doctors Union (protocol number HKSGM-2020AD-Study-protocol-vl-20220211).

### 2.2. Library Preparation and 16S rRNA Sequencing

Fecal specimens were homogenized using a proprietary DNA preservative and subjected to bead beating with 425–600 μm glass beads (Sigma-Aldrich, Burlington, MA, USA) for 1 h in accordance with the manufacturer’s instructions. Microbial DNA was isolated from the fecal specimens using a DNeasy Blood & Tissue Kit (Qiagen, Hilden, Germany). The DNA concentration of each sample was quantified using a Qubit™ dsDNA HS Assay Kit (Life Technologies, Waltham, MA, USA) and a Qubit 3 Fluorometer (Thermo Fisher Scientific, Waltham, MA, USA). An amplicon library was constructed using the 515F(5’-GTGCCAGCMGCCGCGG-3’)/907R(5’-CCGTCAATTTCMTTTRAGTTT-3’) primer pair, targeting the V4-V5 hypervariable region of the 16S rRNA genes in conjunction with adapter sequences and multiplex identifier tags. The 16S rRNA gene sequencing was performed using the Illumina MiSeq platform (Illumina, Inc., San Diego, CA, USA) following the original Earth Microbiome Project Protocols [58]. Index barcodes and adapter sequences were removed from demultiplexed paired-end reads for downstream analysis.

### 2.3. Probiotic Mixture

All AD patients received one sachet daily of a novel synbiotics formula developed by BioMed Microbiome Research Centre (Kid Allergy Formula, BioMed Laboratory Company Limited, Hong Kong) containing a mixture of 6 types of highly effective gastro-resistant probiotics (not less than 1.5 × 10^10^ CFU/sachet at the time of production), and triple prebiotics containing inulin, isomalto-oligosaccharides (IMOs), and fructo-oligosaccharides (FOSs) for 8 weeks. The probiotic mixture was composed of *Lactobacillus rhamnosus* GG, *Lactobacillus acidophilus* GKA7, *Bifidobacterium longum* GKL7, *Lactobacillus plantarum* GKM3, *Bifidobacterium bifidum* GKB2, and *Lactobacillus paracasei* GKS6. *L. rhamnosus* GG and *B. bifidum* formula were shown to reduce the occurrence and recurrence risks of allergies in previous studies. Prebiotics serve as a substrate for probiotics, thereby enhancing their functionality [59].

### 2.4. Quantitative Real-Time PCR

Leftover fecal microbial DNA from 16S rRNA sequencing was retrieved for further analysis. Real-time PCR was carried out with a total volume of 10 μL, containing 5 μL of GoTaq qPCR master mix (Promega Corporation, Madison, WI, USA), 2 μL of DNA template, and 3 μL of primer pair solution (300 nM/reaction). For each run, nuclease-free water (Promega Corporation, Madison, WI, USA) was used as the negative control and melting peaks were used to determine the specificity of the PCR. qPCR was performed in a DNA thermal cycler (QuantStudio 1 Real-Time PCR System, Thermo Fisher Scientific, Waltham, MA, USA).The PCR protocol included an initial denaturation step at 95 °C for 2 min, followed by 40 cycles of 95°C for 15 s and 60 °C for 1 min, and a final dissociation step (95 °C for 15 s, 60 °C for 1 min, followed by a slow ramp to 95 °C). Primer sequences are detailed in Table S1. ∆Ct was calculated as the Ct difference between the respective target and universal bacterial primer.

### 2.5. Bioinformatics Analysis

16S rRNA demultiplexed sequencing data were analyzed using QIIME 2-2023.2 [60], a plugin-based system that integrates various microbiome analysis algorithms and tools. Quality control and denoising of reads were conducted with DADA2 [61] using the q2-dada2 plugin to obtain exact amplicon sequence variants (ASVs) [62]. ASVs were aligned using mafft [63] and a phylogenetic tree was constructed using fastree2 [64] via the q2-phylogeny plugin. Taxonomic annotation of ASVs was performed using the q2-feature-classifier plugin [65] with a pre-trained naive Bayes classifier based on the SILVA v138 taxonomic reference database with 99% similarity [66,67]. Alpha diversity was assessed using six metrics: observed features, Chao1 index (Chao1), ACE index (ACE), Shannon diversity index (Shannon), Simpson index (Simpson), and Faith’s phylogenetic diversity (PD). Beta diversity was assessed based on the Bray–Curtis, cosine, Hamming, Jaccard distance metrics, and the generalized, weighted, weighted normalized, and unweighted UniFrac distance metrics. The difference in gut microbial composition across groups was computed using PERMANOVA test (999 permutations) [68], while the Adonis test was used to investigate the microbial community dissimilarity across responders and time points [69]. Differential abundance analysis was performed using ANCOM with bias correction and repeated measures (ANCOM-BC2) [70]. The co-occurrence/co-exclusion network was derived using the Sparse and Compositionally Robust Inference of Microbial Ecological Networks (SPIEC-EASI) framework [71] using the neighborhood selection framework introduced by Meinshausen and Bühlmann [72].

### 2.6. Statistical Analysis

Statistical analyses and visualization of the results were performed using Python 3.8.13 with the following package versions: numpy 1.22.3, scipy 1.8.0, statsmodels 0.13.2, skbio 0.5.6, matplotlib 3.5.1, and seaborn 0.11.2. Normality assumptions were assessed using D’Agostino and Pearson’s test (implemented in the scipy.stats.normaltest function) and the Shapiro–Wilk test (implemented in the scipy.stats.shapiro function) for parametric tests. Demographic characteristics were evaluated using the non-parametric Mann–Whitney U rank test (implemented in the scipy.stats.mannwhitneyu function) for continuous variables and the Fisher exact test (implemented in the scipy.stats.fisher_exact function) for categorical variables. *p*-value correction was performed using the Benjamini/Hochberg (non-negative) procedure, implemented in the statsmodels.stats.multitest.multipletests function. A *p*-value of less than 0.05 was considered statistically significant unless otherwise specified.

## 3. Results

### 3.1. Study Cohort

A total of 24 subjects aged between 1 and 10 years old with atopic dermatitis were prospectively recruited into the cohort. A summary of the participants’ demographic and disease-related characteristics is presented in Table 1. In brief, the cohort consisted of 58.3% males, and 41.6% of the participants were delivered by Caesarean section. Over 80% of caregivers of the participants reported type-1 hypersensitivity-related comorbidities including allergic rhinitis, asthma, or allergic conjunctivitis. Three participants reported having comorbid constipation, while one participant reported having comorbid diarrhea at baseline. There was one participant aged under 3 years, which is younger than the reported age of gut microbiome maturation [73]. However, we did not exclude the participant from the analysis owing to the small sample size.

The cohort was stratified into subgroups according to the response to the 8-week oral administration of probiotics. There was no statistically significant difference in age (mean age—responders: 9 years, non-responders: 4 years; *p* = 0.0524), sex, or mode of delivery between responders and non-responders. However, age and mode of delivery are well-known confounding factors of gut microbiome analysis; we controlled for age and mode of delivery during subsequent analysis. No adverse effects or discontinuations were reported or recorded throughout the study period.

### 3.2. Effect of 8-Week Probiotic Intake on Gut Microbiome Composition

**Figure 1:**
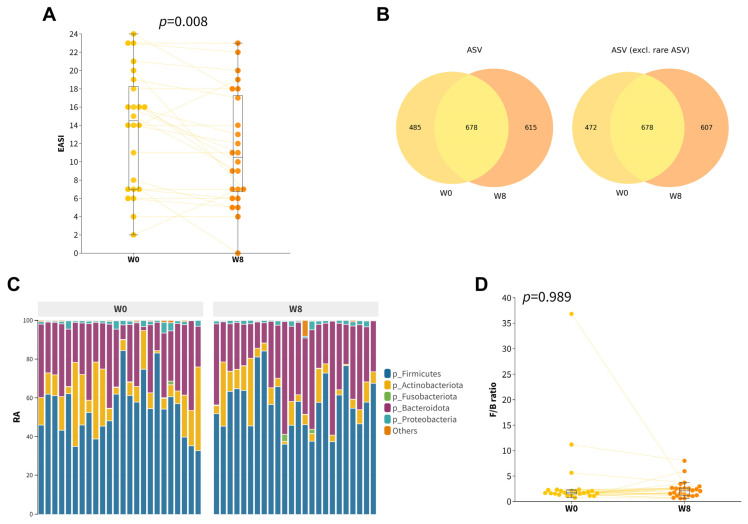
Gut composition profiles of childhood AD participants at baseline and at week 8. (**A**) Boxplot of the EASI scores of the participants. (**B**) Venn diagram of all ASVs (left) and with rare ASVs excluded (right) at baseline and week 8. Rare ASVs were defined as ASVs that occurred in only one of the samples. (**C**) Relative abundance of top five main phyla. (**D**) Boxplot of Firmicutes/Bacteroidetes (F/B) ratio.

Among all participants, there was a modest yet significant improvement in EASI score (∆EASI = −2.3 ± 3.9, *p* = 0.008, Figure 1A) after taking an 8-week course of orally administered probiotics. Our hypothesis posited that the observed improvement in EASI scores could be attributed to the restoration of gut dysbiosis mediated by the synbiotics formula. Therefore, we compared the gut microbiota composition of participants after 8 weeks of probiotic supplementation with their baseline profiles (Figure 1B,C). No significant differences in alpha diversities (Figure S1) and beta diversities (Figure S2) were found. Even though there was an increase in the relative abundance of Firmicutes, together with a decrease in the relative abundances of Actinobacteriota and Desulfobacterota, neither the phylum after multiple testing corrections (Wilcoxon Signed Rank, Benjamini/Hochberg correction) nor the F/B ratio were observed to be significantly different (Figure 1D and Table S2).

**Figure 2:**
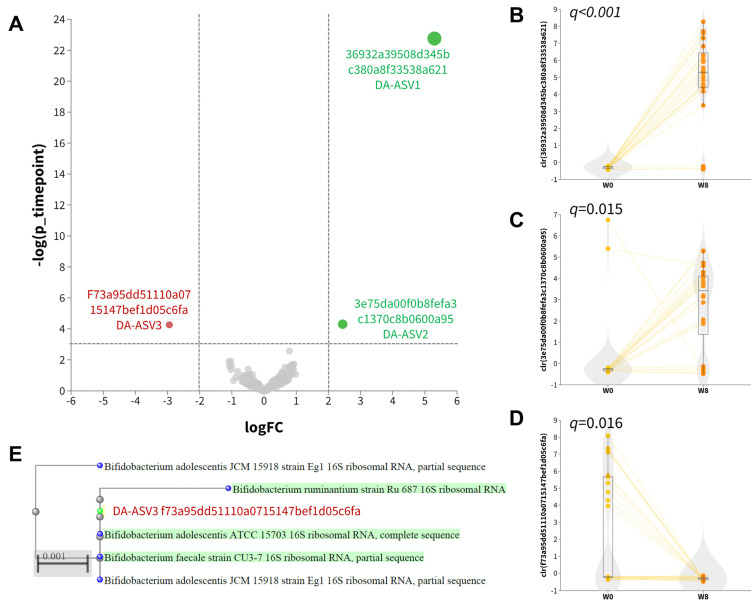
Differentially abundant ASVs. (**A**) Volcano plot of ANCOM-BC2 results. Differentially abundant ASVs indicated in red (downregulated at week 8) and green (upregulated at week 8). (**B**–**D**) Boxplot of center-log-ratio (clr) transformed abundance of differentially abundant ASVs. (**E**) Phylogenetic tree of DA−ASV3 computed by MOLE−BLAST using 16S rRNA sequences as search set with default parameter settings.

An exploratory analysis of differentially abundant units was conducted using ANCOM-BC2 at the ASV level. This analysis identified three differentially abundant ASVs, including two named species (DA−ASV1 and DA−ASV2) and one unnamed species (DA−ASV3), as detailed in Table 2. DA−ASV1 and DA−ASV2 were classified as *Bacteroides fragilis* and *Lactobacillus acidophilus*, respectively (Table S3). Higher (center-log-transformed, clr) relative abundances of DA−ASV1 (Figure 2B; *q* < 0.001) and DA−ASV2 (Figure 2C; *q* = 0.015) were observed at week eight, while a lower relative abundance of DA−ASV3 (Figure 2D) was observed after 8 weeks. MOLE-BLAST was employed to classify the DA−ASV3 unnamed *Bifidobacterium* species as closely related to *Bifidobacterium adolescentis* (Figure 2E). The increase in *Lactobacillus acidophilus* abundance was very likely due to the intake of synbiotics rich in *Lactobacillus*.

### 3.3. Distinctive Changes in the Gut Microbiota among Responders

**Figure 3:**
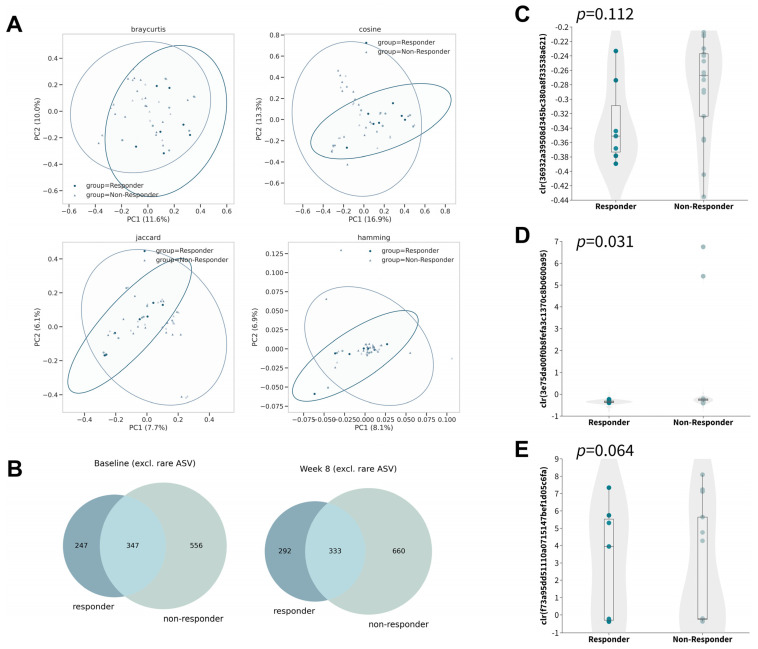
Gut microbiome compositions of responders and non-responders after 8 weeks of synbiotics. (**A**) Principal coordinate analysis based on Bray–Curtis (upper left), cosine (upper right), Jaccard (lower left), and Hamming distance (lower right). (**B**) Venn diagram of ASVs (excluding rare ASVs) at baseline (left) and week 8 (right). (**C**–**E**) Boxplot of center-log ratio (clr)-transformed abundance of DA−ASV at baseline (Mann–Whitney U).

To investigate the differences in the changes in gut microbiota after 8 weeks of oral probiotic intake in the responder subgroup, we further examined the changes in alpha diversities within the subgroup (Figures S3–S5). Although no significant changes were observed in any of the six alpha diversity metrics tested, the Adonis test identified a significant difference in five of the eight beta-diversity metrics by taking responsiveness and timepoint into account (Figure 3A), which indicated the differences in the microbial profiles of responders compared with non-responders (Figure 3B).

Owing to the relatively small sample size in the subgroup, we explored the dissimilarity in the abovementioned differential abundant ASVs without proceeding to subsequent analysis so as to minimize false discovery. In contrast to that of non-responders, the gut microbiota of responders was found to contain less DA−ASV1 (Figure 3C; *p* = 0.112, Mann–Whitney U) and DA−ASV2 (Figure 3D; *p* = 0.031, Mann–Whitney U) and more DA−ASV3 (Figure 3E; *p* = 0.064, Mann–Whitney U) at baseline with marginal statistical significance, while no statistical significance was discovered at week eight (Figure S6). Interestingly, the observation was opposite to the direction of the respective log fold change. This may imply that people who have a lower abundance of DA−ASV1 and DA−ASV2, and a higher abundance of DA−ASV3, would be more likely to respond to probiotics for the control of atopic dermatitis.

### 3.4. The Dynamics of Probiotic Species in Relation to Probiotic Intake Responsiveness

**Figure 4:**
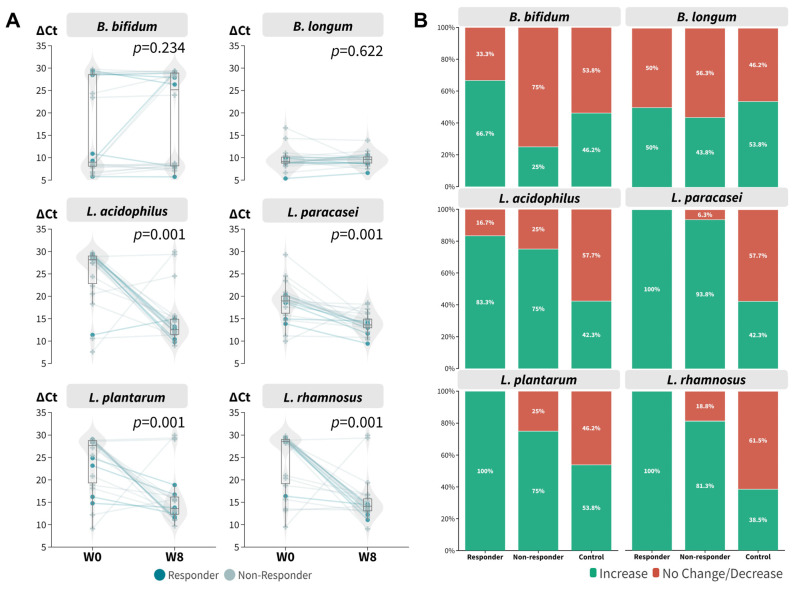
Real-time PCR of beneficial bacteria contained in the synbiotics mixture. (**A**) ∆Ct at baseline and week 8 (signed rank test regardless of responsiveness). (**B**) Proportion of participants within responsiveness subgroup who achieved increase in relative abundance of respective bacterial targets.

With the encouraging results with respect to the increased relative abundance of *L. acidophilus* after 8 weeks of probiotic intake, we further verified the findings using qPCR (Table S4) because it is well known that 16S targeted sequencing has a limited resolution at the species level. All probiotic species contained in the mixture were tested using the remaining DNA extract. It is evident that the ∆Ct values of *L. acidophilus* (*p* = 0.001, Wilcoxon signed rank), *L. paracasei* (*p* = 0.001, Wilcoxon signed rank), *L. plantarum* (*p* = 0.001, Wilcoxon signed rank), and *L. rhamnosus* (*p* = 0.001, Wilcoxon signed rank) were decreased at week eight regardless of responsiveness (Figure 4A), which corresponded to an increase in relative abundance. The results for *B. bifidum* and *B. longum* were mixed. Overall, responders were found to have a relatively higher relative abundance of probiotic species (Figure 4B). We also incorporated a historical placebo cohort as a control for comparison. The synbiotics formula boosted the relative abundance of probiotic species to a greater extent compared to the control group.

### 3.5. Distinctive Functional Abundance Profile among Responders

**Figure 5:**
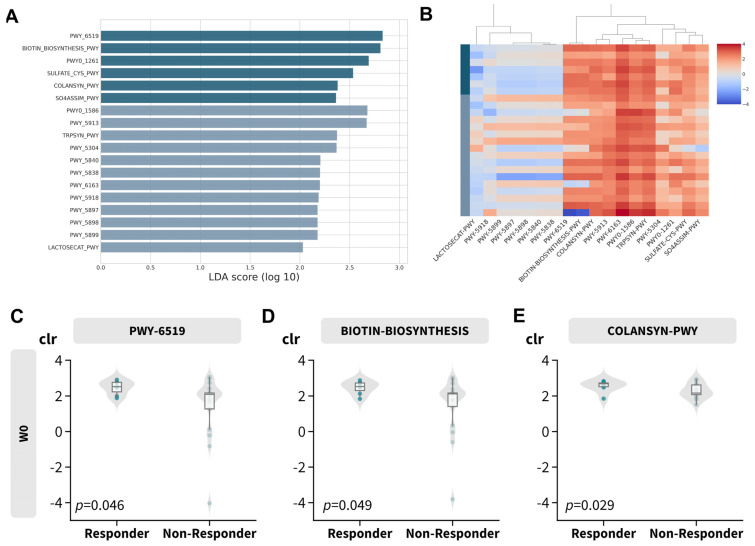
Predicted MetaCyc pathway abundance. (**A**) Log LDA score of differentially abundant MetaCyc pathways. (**B**) Heatmap of center−log−ratio (clr) transformed abundance of differentially abundant MetaCyc pathways. (**C**–**E**) Boxplot of center−log−ratio (clr) transformed abundance of selected differential abundant MetaCyc pathways.

To investigate the biological implications of the gut microbiome profile, functional abundance was inferred using PICRUSt2 in conjunction with LefSe. This analysis identified 18 discriminative features with an absolute LDA score greater than 2, as depicted in Figure 5A,B and detailed in Table 3. Most of the features over-represented in the responder subgroup (Figure S7) were related to vitamin B synthesis, such as biotin (Figure 5C,D; PWY-6519 and BIOTIN-BIOSYNTHESIS) and colonic acid (COLANSYN-PWY). However, vitamin K or menaquinol-related pathways (including PWY-5840, PWY-5838, PWY-5897, PWY-5898, and PWY-5899) and L-tryptophan synthesis were disproportionately abundant in non-responders (TRPSYN-PWY and PWY-6163).

Notably, peptidoglycan recycling (PWY0-1261; leading to decreased expression of peptidoglycan) was over-represented in responders, while peptidoglycan maturation (PWY0-1586; leading to increased expression of peptidoglycan) was over-represented in non-responders. Signature fragments of peptidoglycan are known to trigger pro-inflammatory responses [74], which may explain the responsiveness in relation to the gut microbial profile.

## 4. Discussion

Despite the recent advancements in therapeutic options in the management of atopic dermatitis, including the use of biologics and small molecules [75,76], recurrent disease is commonplace in AD patients, yielding poor treatment satisfaction, frustration, and anxiety in patients and their caregivers [77]. In addition, the journey of children with AD is often complicated by their reluctance to use corticosteroids [78]. Therefore, it is crucial to seek alternative options to complement existing management plans. Probiotic supplementation has emerged as an attractive choice with the substantial recognition of the gut–skin axis and the pivotal role of the gut microbiome in immunity maturation [79]. In our study, we explored the gut microbial dynamics upon probiotic intake and clarified the taxonomical and functional alteration of the gut microbiome profile among responders in a pediatric atopic dermatitis cohort. To our knowledge, this represents the first study to investigate the dynamics of the gut microbiome in response to probiotic supplementation in a pediatric atopic dermatitis cohort in Hong Kong. We posit that the inclusion of gut microbiome data from southern Chinese patients could enhance our understanding of the convoluted reciprocity between the gut microbiome and host health.

We reported three significant differentially abundant ASVs (DA−ASVs) after 8 weeks of oral probiotic administration without significant changes in conventional alpha diversities, beta diversities, and taxonomic analysis. It is expected that the impact of probiotics on the gut microbiota would be reflected to a larger extent on the ASV level rather than structural changes, because the gut microbiota is reported to be resilient [80,81,82,83], and this pilot cohort may not be suitable for illustrating the subtle differences in the gut flora. Among the DA−ASVs identified, *B. fragilis* is reportedly associated with childhood eczema [30,32]. The bacterial polysaccharide produced by *B. fragilis* is known to exert anti-inflammatory properties via inducing IL-10 secretion, balancing Th1/Th2 populations, and modulating systemic T cell deficiencies [84,85,86]. *L. acidophilus* has also been repeatedly shown to suppress inflammation and improve atopic dermatitis symptoms [47,87,88,89,90]. However, one of the unnamed DA−ASVs that is closely related to *B. adolescentis* was depleted after probiotic intake. This observation was also made in a recent 4-month prospective study of infant eczema in Hong Kong [30], and in contrast to a previous study in Swedish infants with atopic dermatitis [33]. This further reinforces and justifies the need to conduct more studies with diverse ethnicities and across a wide range of geographical locations to gain a better understanding of the complex interplay between microbes and hosts.

We also noted that participants with a lower relative abundance of *B. fragilis* or *L. acidophilus* and a higher relative abundance of DA−ASV3 (*B. adolescentis*) may be more likely to benefit from the synbiotics formula. As *L. acidophilus* is one of the components of the synbiotics mixture, we decided to conduct real-time PCR to examine the relative abundances of the probiotic species contained in the mixture. It is evident that probiotic intake increased the relative abundance of beneficial bacteria reaching statistical significance in four out of six bacterial targets tested. Specifically, no effect on *B. longum* was observed, which could likely result from its high abundance at baseline compared to other bacterial targets. This echoes previous landmark findings showing that the baseline gut microbiome profile may play a decisive role in determining a host’s response towards probiotics [57,91]. Responders were found to have a higher relative abundance of probiotic species when compared to non-responders. It is possible that attaining adequate levels of intestinal probiotic species is necessary for an optimal clinical response. This may explain the inconsistent response to the use of oral probiotics in AD in previous clinical studies. Monitoring the adequacy of probiotic therapy with microbiome-based testing with a defined level endpoint may be a potential option to optimize the therapeutic outcome when using probiotic therapy in AD.

To gain physiological insights from the gut microbiome profile, functional abundances were investigated. Vitamin B7 (biotin) and B12 (cobalamin) synthesis was predicted to be over-represented in responders, which could imply that more vitamin B7 and vitamin B12 have been absorbed and circulated in responders’ bodies [92,93,94]. Several studies suggested that vitamin B might alleviate eczema symptoms [95,96,97,98]. It is also important to highlight that responders exhibited a high prevalence of peptidoglycan recycling, whereas peptidoglycan maturation was predominantly observed in non-responsive participants in line with the pro-inflammatory properties of the signature fragments of peptidoglycan [74]. The abovementioned observation may provide valuable insights into the correlation between the responsiveness of subjects and their respective gut microbial profiles. Vitamin B deficiency has long been known to have a dermatological manifestation [99]. For example, biotin deficiency can lead to skin rash or eczema [100,101,102]. The serum vitamin B7 level has been shown to be associated with cedar pollinosis [103], while topical vitamin B12 has been shown to reduce the severity of atopic dermatitis [104,105,106]. Although blood samples were not included in the current study design, serum vitamin B7 and B12 levels would be valuable measurements in future investigations to gain a deeper understanding of the mechanism of probiotics in pediatric AD.

In summary, our findings show the alteration of the gut microbiome due to probiotic intake in southern Chinese pediatric atopic dermatitis patients. *B. fragilis* and *L. acidopilus* may be crucial microbial drivers in atopic dermatitis development, and probiotics could potentially enhance their impact. We have also provided additional evidence to support the notion of differential probiotic colonization in a personalized manner. Our study was constrained by a limited sample size and the inclusion of only patients with mild to moderate disease severity. Well-designed and powered prospective clinical trials would be warranted to explore the efficacy of probiotics as long-term adjuvant maintenance therapy in AD patients with different disease severities. In spite of this, our study suggests the possibility and provides a scientific basis for the incorporation of probiotic treatment in the management of childhood AD, with the use of microbiome-based testing to assess the therapeutic adequacy endpoint.

## 5. Conclusions

We revealed significant alterations in the composition of the gut microbiome of (1) children with AD taking 8 weeks of probiotics and (2) responders consisting of a local cohort of southern Chinese patients. The crucial microbial drivers *B. fragilis* and *Lactobacillus* were restored at week eight. The gut microbiome profile before and after treatment may predict probiotic responsiveness, and this study could provide an important clue regarding the implication of gut-microbiome-based testing and therapies in the management of children with atopic dermatitis.

## Figures and Tables

**Table 1:** Baseline demographic of participants.

	Responder ^&^ (*n* =7)	Non-Responder (*n* = 17)	*p*-Value
Sex, No. (%)			0.3926
Male	3 (42.9)	11 (64.7)	
Female	4 (57.1)	6 (35.3)	
Age, mean (SD) (range), y	9 [3,4,5,6,7,8,9,10,11,12,13]	4 [1,2,3,4,5,6,7,8,9,10]	0.0524
Mode of delivery, No. (%)			
Vaginal	6 (85.7)	8 (47.1)	0.1718
Caesarean	1 (14.3)	9 (52.3)	
Other allergic comorbidities, No. (%)	6 (85.7)	14 (82.4)	>0.9999
Gastrointestinal, No. (%)			
Constipation	0 (0)	3 (17.6)	0.5296
Diarrhea	1 (14.3)	0 (0)	0.2917
EASI, mean (SD) ^^^	16.3 (5.2)	12.5 (6.8)	0.1707

^&^ Those who achieved ∆EASI 20% were regarded as responders. ^^^ EASI, Eczema Area and Severity Index.

**Table 2:** Differentially abundant ASVs (taxon assigned by q2-feature-classifier) identified by ANCOM-BC2 after the 8-week course of orally administered probiotics.

Feature ID	Taxon	LogFC	SE	W	*p*	*q*
36932a39508d345bc380a8f33538a621	g_Bacteroides; s_Bacteroides_fragilis	5.297	0.530	9.986	<0.0001	<0.0001
3e75da00f0b8fefa3c1370c8b0600a95	g_Lactobacillus; s_Lactobacillus_acidophilus	2.444	0.603	4.056	0.0001	0.0145
f73a95dd51110a0715147bef1d05c6fa	g_Bifidobacterium; s_metagenome	−2.944	0.730	−4.035	0.0001	0.0158

**Table 3:** Differentially abundant MetaCyc pathways and corresponding LDA scores; and *p*-value inferred using PICRUSt2 and LefSe.

BioCyc ID	MetaCyc Pathway Name	Group	Log LDA	*p*-Value
PWY_6519	8-amino-7-oxononanoate biosynthesis I	Responder	2.8318	0.0459
BIOTIN_BIOSYNTHESIS_PWY	Biotin biosynthesis I	Responder	2.8097	0.0485
PWY0_1261	Peptidoglycan recycling I	Responder	2.6914	0.0057
SULFATE_CYS_PWY	Superpathway of sulfate assimilation and cysteine biosynthesis	Responder	2.5342	0.0207
COLANSYN_PWY	Colanic acid building blocks biosynthesis	Responder	2.3794	0.0294
SO4ASSIM_PWY	Assimilatory sulfate reduction I	Responder	2.3628	0.0111
PWY0_1586	Peptidoglycan maturation (meso-diaminopimelate containing)	Non-Responder	2.6783	0.0435
PWY_5913	Partial TCA cycle (obligate autotrophs)	Non-Responder	2.6711	0.0412
TRPSYN_PWY	L-tryptophan biosynthesis	Non-Responder	2.3738	0.0005
PWY_5304	Superpathway of sulfur oxidation (Acidianus ambivalens)	Non-Responder	2.3687	0.0412
PWY_5840	Superpathway of menaquinol-7 biosynthesis	Non-Responder	2.2056	0.0459
PWY_5838	Superpathway of menaquinol-8 biosynthesis I	Non-Responder	2.2025	0.0435
PWY_6163	Chorismate biosynthesis from 3-dehydroquinate	Non-Responder	2.2017	0.0435
PWY_5918	Superpathway of heme b biosynthesis from glutamate	Non-Responder	2.1888	0.0195
PWY_5897	Superpathway of menaquinol-11 biosynthesis	Non-Responder	2.1782	0.0485
PWY_5898	Superpathway of menaquinol-12 biosynthesis	Non-Responder	2.1782	0.0485
PWY_5899	Superpathway of menaquinol-13 biosynthesis	Non-Responder	2.1782	0.0485
LACTOSECAT_PWY	Lactose degradation I	Non-Responder	2.0306	0.0369

## Data Availability

The raw sequence data are available in NCBI (BioProject PRJNA982573). Due to limitations of the consent and concerns regarding sensitive data, access to the metadata and qPCR data is restricted and available only upon reasonable request to the corresponding authors.

## Supplementary Materials

The following supporting information can be downloaded at https://www.mdpi.com/article/10.3390/microorganisms11092175/s1, Supplementary Figure S1 Boxplot of alpha diversity metrics at baseline and week 8 (Wilcoxon signed rank): (A) ACE, (B) Chao1, (C) Faith’s phylogenetic diversity (Faith PD), (D) observed features, (E) Shannon’s index, and (F) inverse Simpson; Supplementary Figure S2. Gut composition profile at baseline and week 8. Principal coordinate analysis biplot based on (A) Bray–Curtis, (B) Jaccard, (C) cosine, (D) Hamming, (E) unweighted UniFrac, (F) weighted UniFrac, (G) generalized UniFrac, and (H) weighted normalized UniFrac (Adonis); Supplementary Figure S3. Boxplot of alpha diversity metrics at baseline and week 8 stratified by responsiveness (Wilcoxon signed rank): (A) ACE, (B) Chao1, (C) Faith’s phylogenetic diversity (Faith PD), (D) observed features, (E) Shannon’s index, and (F) inverse Simpson; Supplementary Figure S4. Boxplot of alpha diversity metrics at baseline stratified by responsiveness (Mann–Whitney U): (A) ACE, (B) Chao1, (C) Faith’s phylogenetic diversity (Faith PD), (D) observed features, (E) Shannon’s index, and (F) inverse Simpson; Supplementary Figure S5. Boxplot of alpha diversity metrics at week 8 stratified by responsiveness (Mann–Whitney U): (A) ACE, (B) Chao1, (C) Faith’s phylogenetic diversity (Faith PD), (D) observed features, (E) Shannon’s index, and (F) inverse Simpson; Supplementary Figure S6. Boxplot of clr-transformed abundance of the differentially abundant ASVs identified by ANCOM-BC2 at week 8. (A) DA−ASV1, (B) DA−ASV2, and (C) DA−ASV3; Supplementary Figure S7. Over-represented functional MetaCyc pathways. (A) Violin plot of over-represented pathways stratified by responsiveness. Boxplots of clr-transformed predicted abundances of (B) PWY-6519, (C) BIOTIN-BIOSYNTHESIS, and (D) COLANSYN-PWY. Supplementary Table S1. qPCR primer sequences of beneficial bacteria; Supplementary Table S2. Adjusted *p*-value of the relative abundance at phylum level; Supplementary Table S3. Confidence of taxonomic assignment of differentially abundant ASVs identified by ANCOM-BC2; Supplementary Table S4. Fisher’s exact test result of the change in abundance stratified by responsiveness.
