# Supplementary figures and images for: Improvements in Gut Microbiome Composition Predict the Clinical Efficacy of a Novel Synbiotics Formula in Children with Mild to Moderate Atopic Dermatitis
Source: Microorganisms. 2023 Aug 28;11(9):2175. doi: 10.3390/microorganisms11092175 (PMC10536305; doi:10.3390/microorganisms11092175)

Figure S1

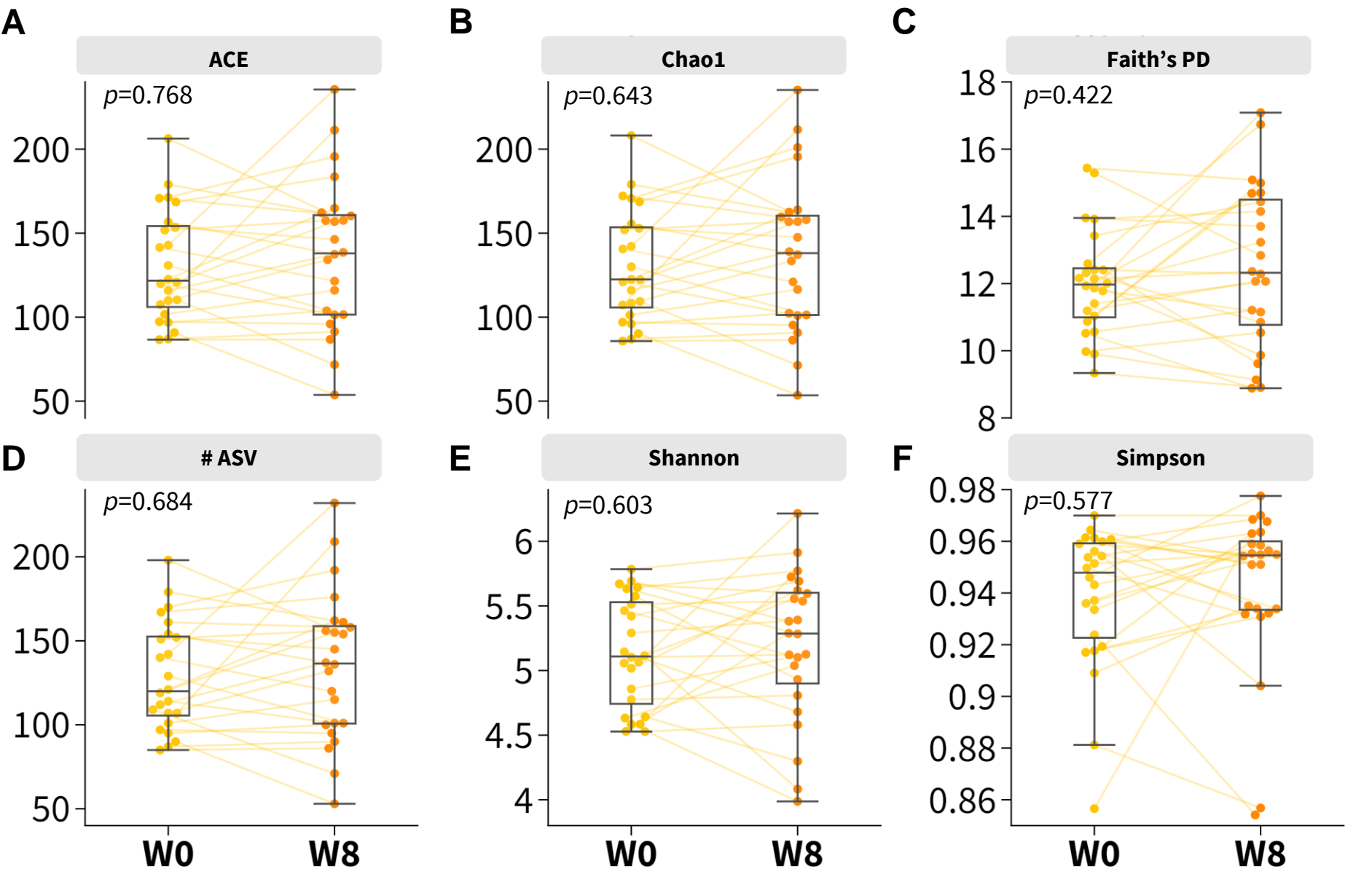

Figure S2

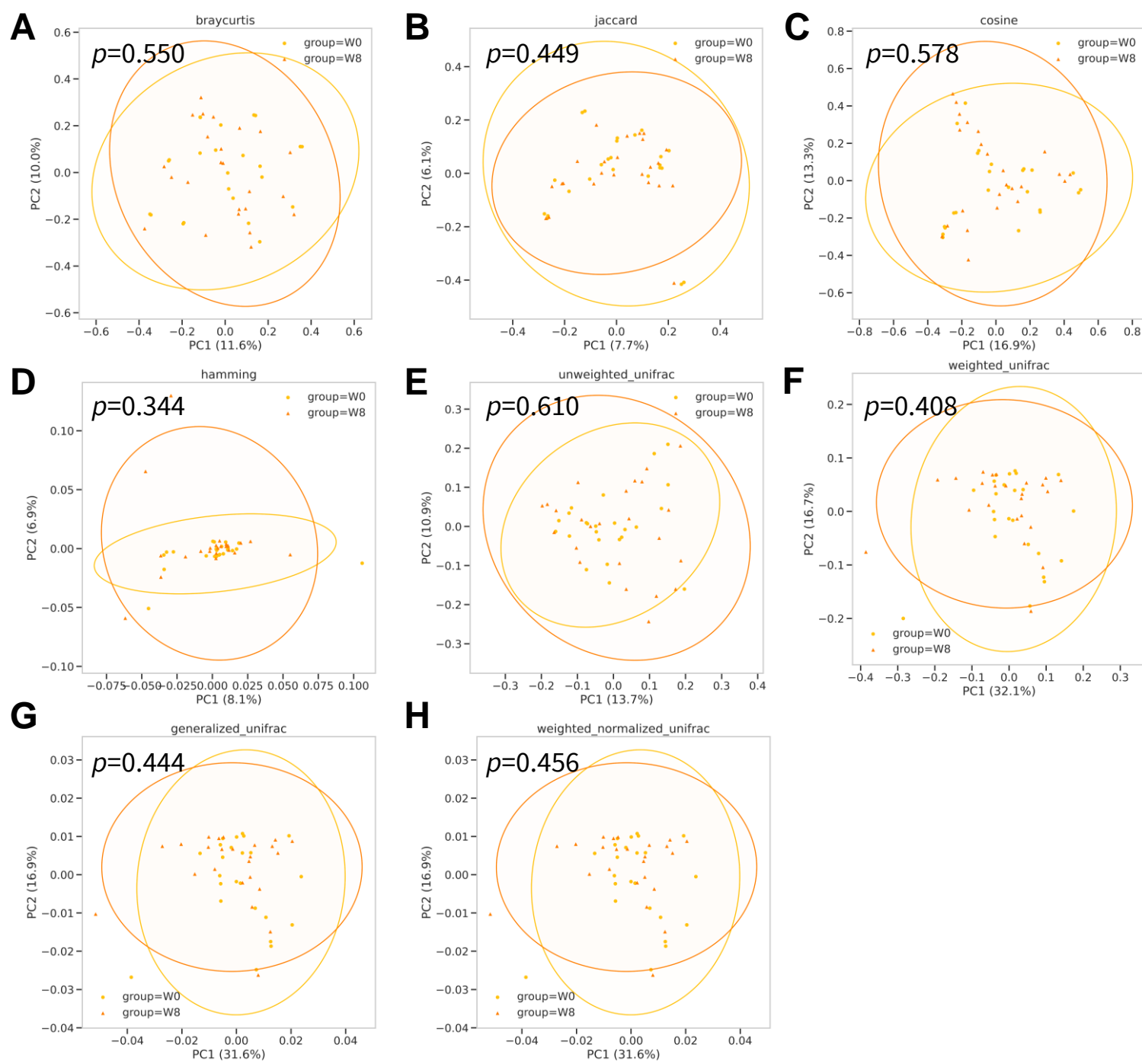

Figure S3

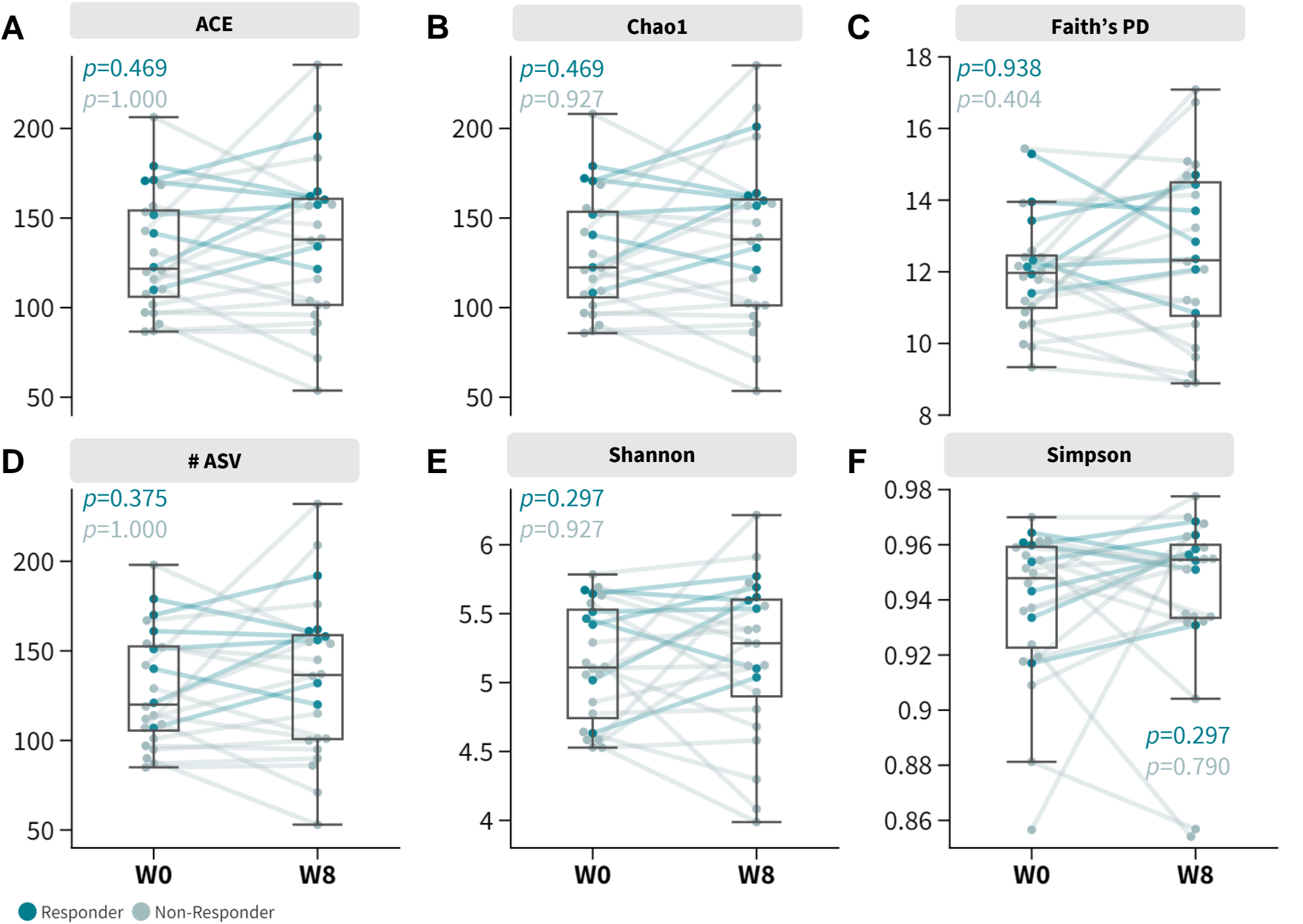

Figure S4

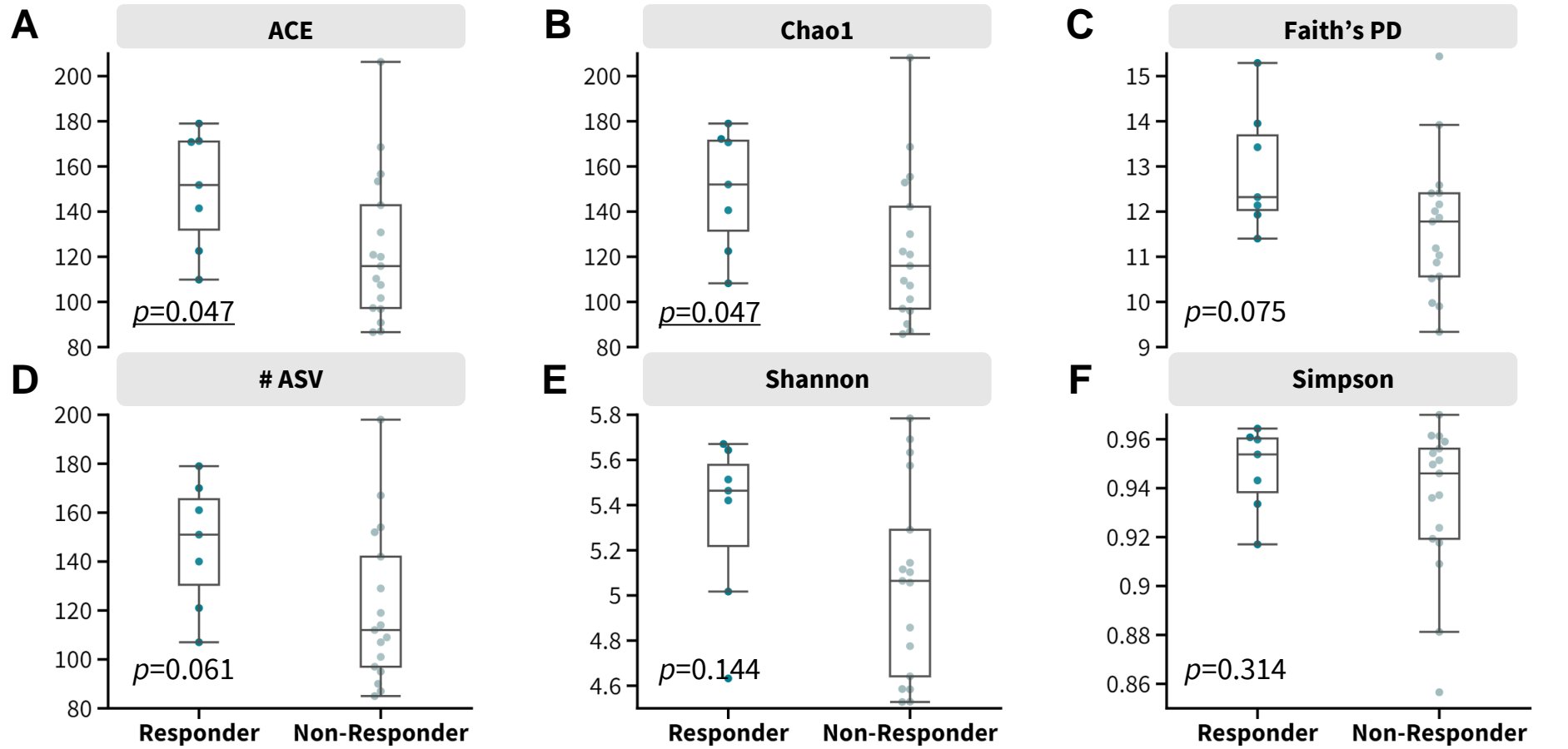

Figure S5

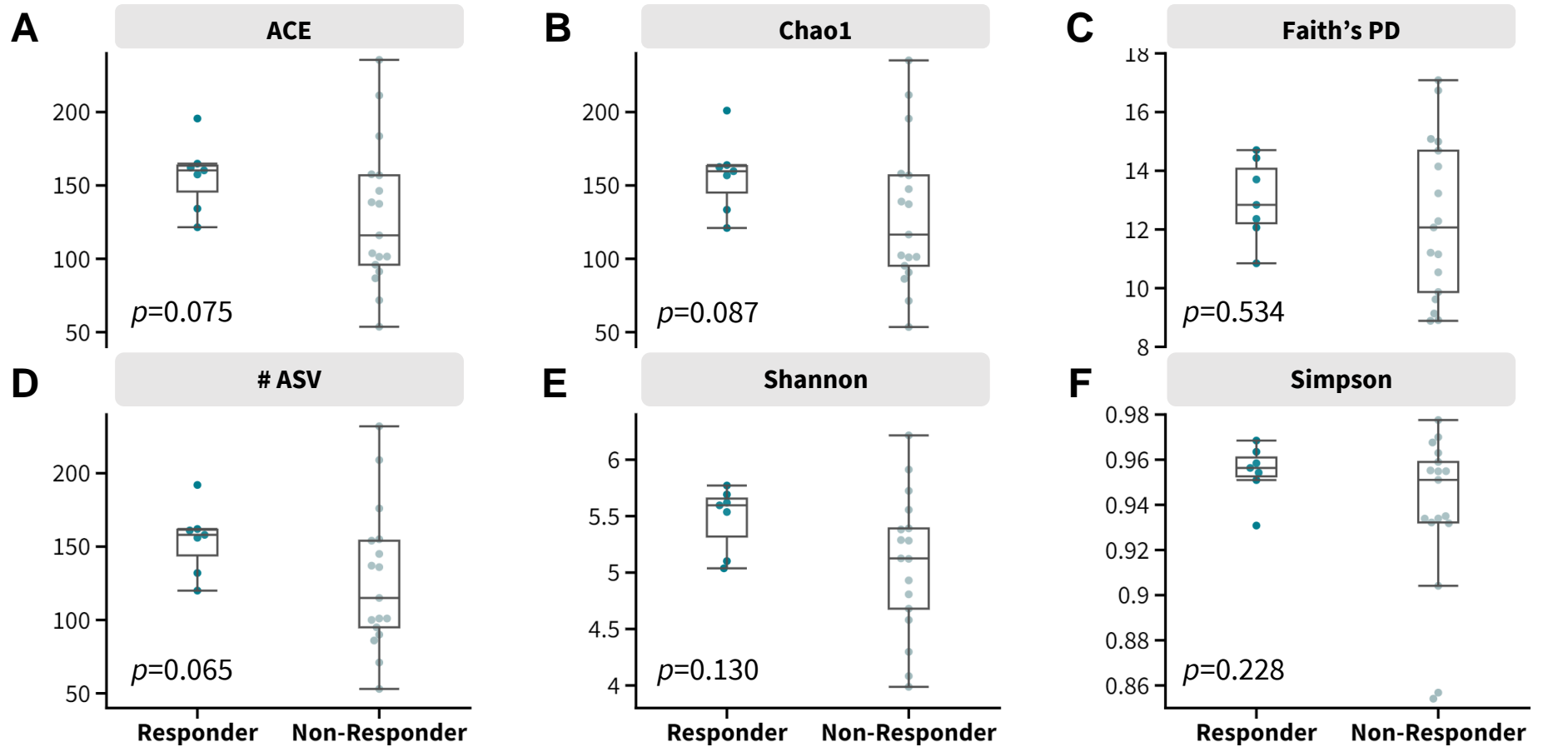

Figure S6

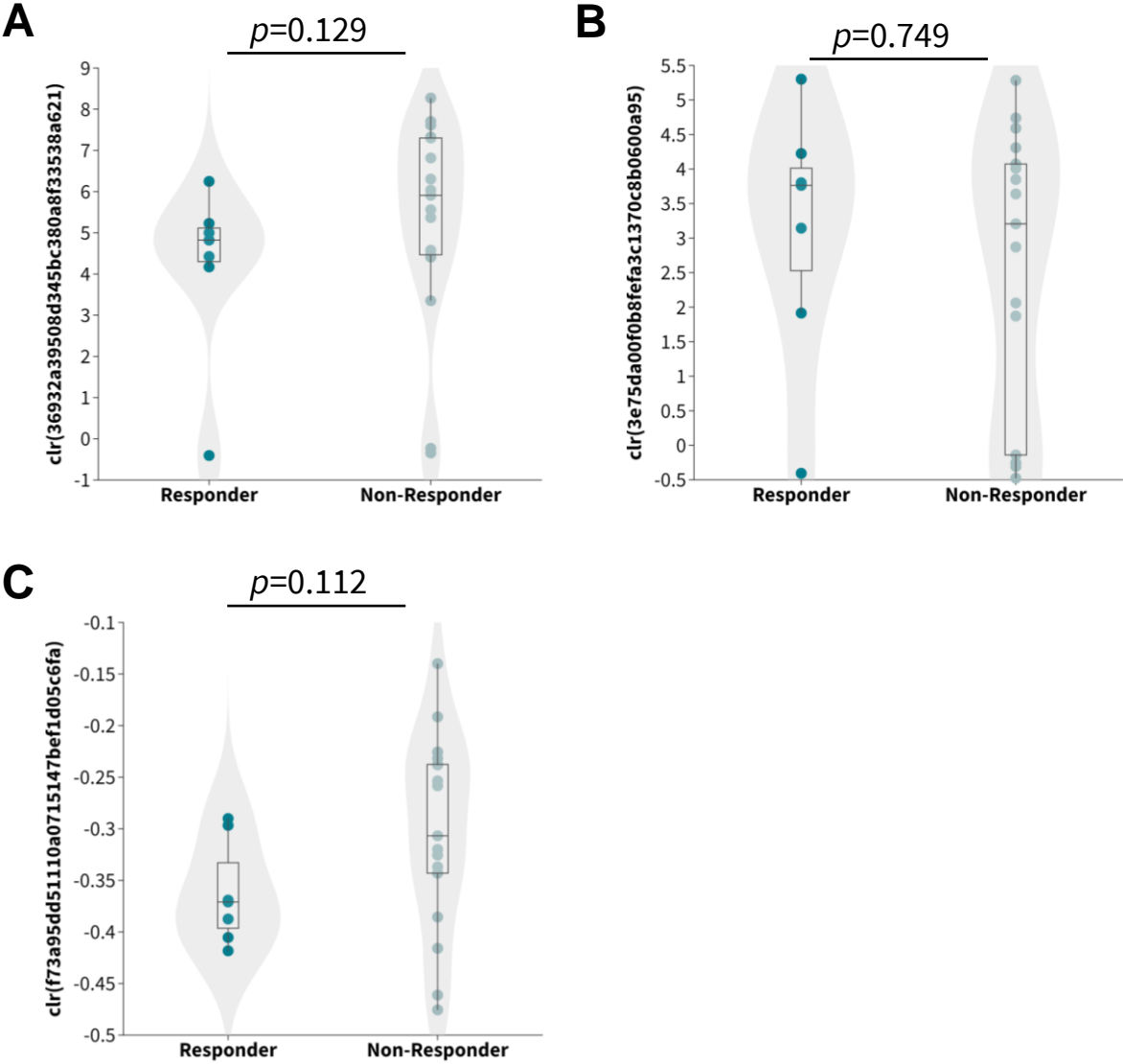

Figure S7

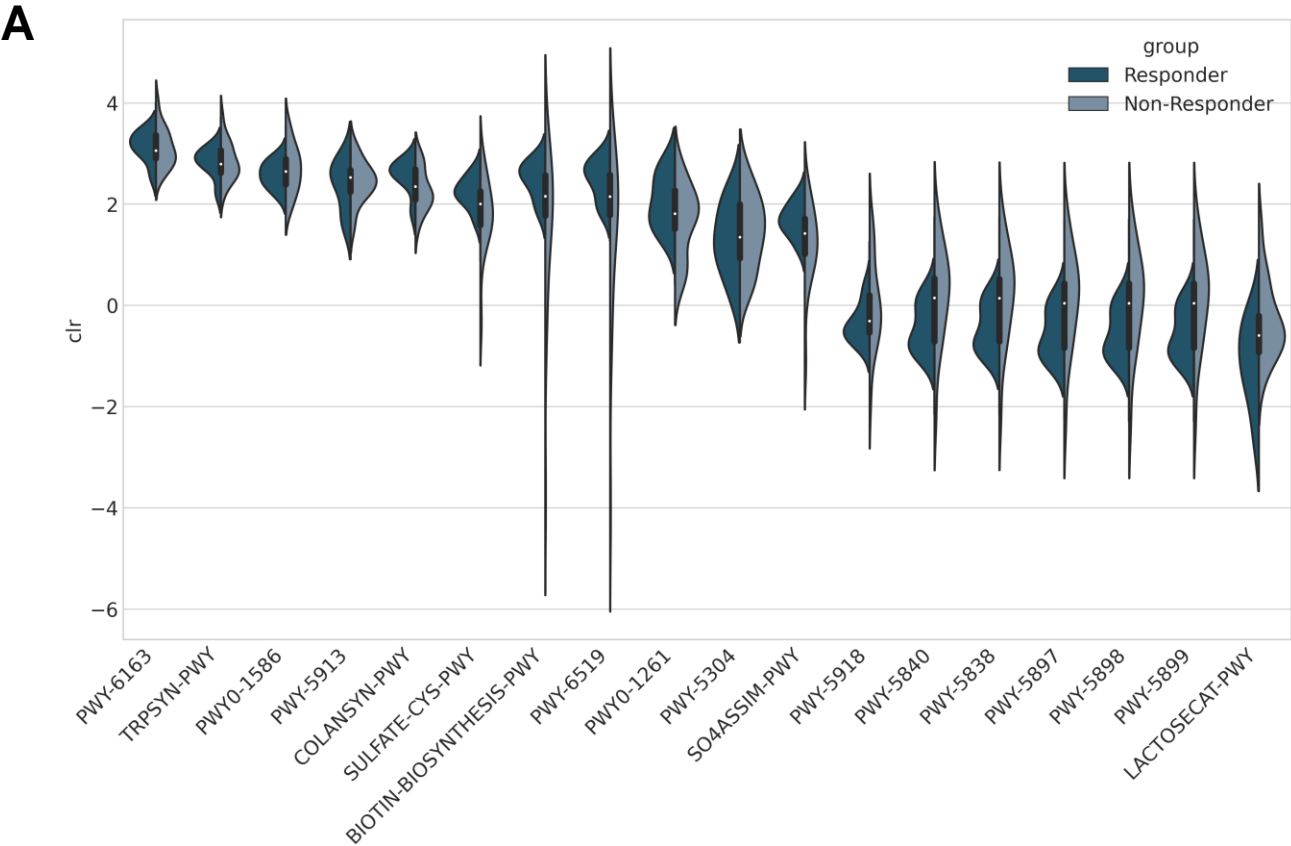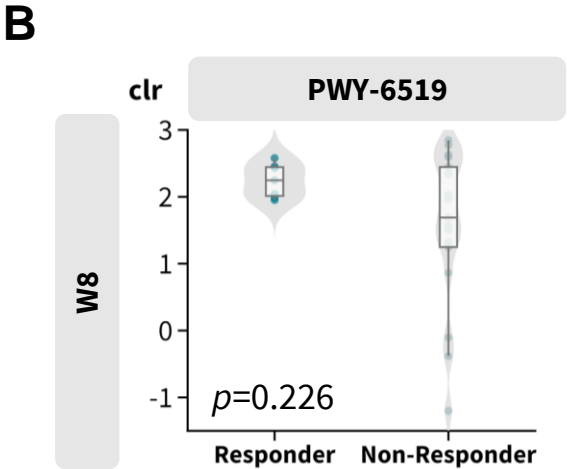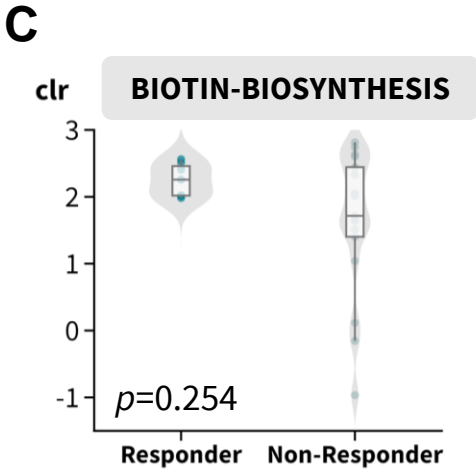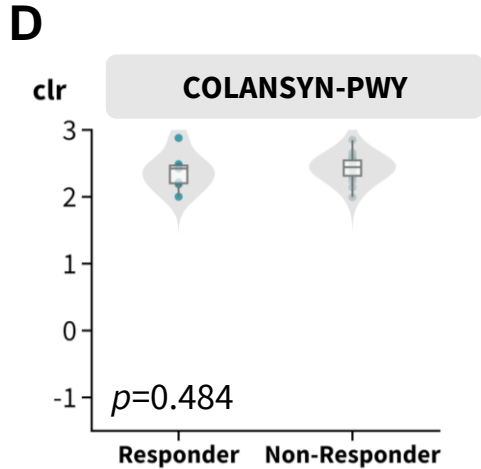

Supplement: Supplementary file 1 [file microorganisms-11-02175-s001.zip › suppl-figures_20230605.pdf]
